# Simulated Microgravity Exerts an Age-Dependent Effect on the Differentiation of Cardiovascular Progenitors Isolated from the Human Heart

**DOI:** 10.1371/journal.pone.0132378

**Published:** 2015-07-10

**Authors:** Tania I. Fuentes, Nancy Appleby, Michael Raya, Leonard Bailey, Nahidh Hasaniya, Louis Stodieck, Mary Kearns-Jonker

**Affiliations:** 1 Department of Pathology and Human Anatomy, Loma Linda University School of Medicine, Loma Linda, California, United States of America; 2 Department of and Cardiothoracic Surgery, Loma Linda University School of Medicine, Loma Linda, California, United States of America; 3 BioServe Space Technologies, Department of Aerospace Engineering Sciences, University of Colorado, Boulder, Colorado, United States of America; San Raffaele Pisana, ITALY

## Abstract

Microgravity has a profound effect on cardiovascular function, however, little is known about the impact of microgravity on progenitors that reside within the heart. We investigated the effect of simulated microgravity exposure on progenitors isolated from the neonatal and adult human heart by quantifying changes in functional parameters, gene expression and protein levels after 6-7 days of 2D clinorotation. Utilization of neonatal and adult cardiovascular progenitors in ground-based studies has provided novel insight into how microgravity may affect cells differently depending on age.

Simulated microgravity exposure did not impact AKT or ERK phosphorylation levels and did not influence cell migration, but elevated transcripts for paracrine factors were identified in neonatal and adult cardiovascular progenitors. Age-dependent responses surfaced when comparing the impact of microgravity on differentiation. Endothelial cell tube formation was unchanged or increased in progenitors from adults whereas neonatal cardiovascular progenitors showed a decline in tube formation (p<0.05). Von Willebrand Factor, an endothelial differentiation marker, and MLC2v and Troponin T, markers for cardiomyogenic differentiation, were elevated in expression in adult progenitors after simulated microgravity. DNA repair genes and telomerase reverse transcriptase which are highly expressed in early stem cells were increased in expression in neonatal but not adult cardiac progenitors after growth under simulated microgravity conditions. Neonatal cardiac progenitors demonstrated higher levels of MESP1, OCT4, and brachyury, markers for early stem cells. MicroRNA profiling was used to further investigate the impact of simulated microgravity on cardiovascular progenitors. Fifteen microRNAs were significantly altered in expression, including microRNAs-99a and 100 (which play a critical role in cell dedifferentiation). These microRNAs were unchanged in adult cardiac progenitors.

The effect of exposure to simulated microgravity in cardiovascular progenitors is age-dependent. Adult cardiac progenitors showed elevated expression of markers for endothelial and cardiomyogenic differentiation whereas neonatal progenitors acquired characteristics of dedifferentiating cells.

## Introduction

Microgravity, as experienced by humans when in space, affects cardiovascular function resulting in post-flight orthostatic intolerance, cardiac atrophy, and heart rhythm disturbances [1]. However, little is known about the impact of altered gravitational force on cardiac progenitors that normally reside within the heart. Simulations of gravitational changes while here on earth have demonstrated that cell types, such as cardiomyocytes, are force-sensitive. This may be due to mechanosensors which operate within mechanotransduction pathways that alter cell function upon exposure to changes in the force of gravity [2]. In mesenchymal stem cells, hypergravity increased differentiation into cardiomyocytes and osteoblasts [3] whereas simulated low-gravity inhibited osteogenesis [4] and resulted in higher levels of adipogenesis [3, 5, 6]. Exposure of other stem cell types to simulated microgravity such as embryonic stem cells [7], umbilical cord blood stem cells [8], adipose-derived stem cells [9], liver stem cells [10], and cancer stem cells [11] have established a link between gravitational force and changes in cell identity, either towards stemness or differentiation.

Stem cell responses to simulated microgravity may be influenced by age. With age, the regenerative capacity of cardiovascular progenitors found within the heart decreases [12]. We have previously reported that Isl-1 positive cardiovascular progenitors isolated from the neonatal and adult heart exhibit age—dependent disparities in functional parameters such as cell cycle progression and invasion which may stem from underlying differences in gene and microRNA expression [13]. Determining the effect of simulated microgravity on resident cardiac progenitor cell function *in vitro*, and whether this effect is dependent on age would have implications not only for space flight, but would also provide evidence as to whether alterations in gravitational force could be utilized as a therapeutic for improved cardiovascular stem cell function.

In this report, we characterized the effect of simulated microgravity on both neonatal and adult cardiovascular Isl-1+ progenitor cell clones by culturing them in a 2D clinostat which minimizes the gravitational force by rotating cells perpendicular to the pull of gravity. Functional parameters including cell cycle progression, migration, cell differentiation into endothelial and cardiomyogenic lineages, as well as gene and protein expression changes after 6–7 days in simulated microgravity were assessed. Our approach allowed us to define both age-related and overall responses of endogenous cardiovascular progenitors to simulated microgravity conditions.

## Materials and Methods

### Ethics Statement/Cell Isolation and Expansion

The Institutional Review Board of Loma Linda University approved the protocol for use of tissue that was discarded during cardiovascular surgery, without identifiable private information, for this study with a waiver of informed consent. In brief, right atrial cardiac tissue from human neonates (<1 month old) and adults (57–75 years old), was cut into small clumps (approximately 1 mm^3^) and collagenase digested (Roche Applied Science, Indianapolis, IN) for approximately 2 hours at 37 degrees at a proportion of 1:2.5 tissue volume vs. collagenase. This solution was then passed through a 40 μm cell strainer to isolate cardiac progenitors [14]. Resulting cells were cloned by limiting dilution at a concentration of 0.8 cells per well to create clonal populations which were expanded for further study.

### Simulated Microgravity

Isl-1+ cardiac stem cell clones from both the neonatal and adult heart were cultured for 6–7 days in a 2D clinostat (Bioserve, Boulder, Colorado). A 2D clinostat simulates the absence of gravity by maintaining cells in constant rotation perpendicular to the force of gravity. Cells were seeded at a density of 200,000 cells per Opticell using both inner surfaces for cell growth or 100,000 cells per Biocell with one surface for cell growth, gassed with a mixture of 5% CO_2_, 95% air, then subjected to clinorotation. The Biocell is centered within the rotating chamber. The width provides a cell growth area of 5.5cm. The rotation rate is 3.94+/-0.01 rotations per minute. The cells farthest from the center will experience the greatest centrifugal forces as a consequence of the rotation. The relative centrifugal force on these cells is <0.5mG.

Once CPCs reached confluency within the Opticell or Biocell (6 or 7 days), they were trypsinized, counted, and used for subsequent experiments. Controls were similarly seeded and grown under static conditions within a 5% CO_2_ cell culture incubator for a matched period of time.

### Migration Assay

After exposure to 6–7 days of clinorotation, cells were trypsinized counted, resuspended in starving medium, and plated in the top chamber of a 96-well transwell migration assay (Corning, Union City, CA) with 8 micron pores. The transwell migration assay was performed according to manufacturer’s instructions (Corning, Union City, CA). In brief, cells were plated at a density of 50,000 cells per well in the top transwell chamber and growth medium supplemented with 100ng/mL of SDF-1α was used in the bottom chamber as a chemoattractant (Life Technologies, Grand Island, NY). After 6 hours, migrated cells in the bottom chamber were stained using Calcein AM (Fisher Scientific Pittsburg, PA) and quantified using a FLX800 fluorescent plate reader (Bio-Tek, Winooski, VT).

### Flow Cytometry

After exposure to clinorotation, MAPK signaling was assessed by measuring the phosphorylation of ERK and AKT by flow cytometry. Quantification of phosphorylated protein levels by flow cytometry correlates with results obtained by western blot [15–17], and allows us to determine relative mean fluorescence intensity in individual cells [18]. In brief, cells were fixed with 4% paraformaldehyde, permeabalized using methanol, and stained using antibodies to phosphorylated ERK 1/2 (Thr202/Tyr204, Cell Signaling Technology, Danvers, MA) at a 1/200 dilution, and p-AKT (Ser473, Cell Signaling Technology, Danvers, MA) at a 1/100 dilution, FITC goat anti-rabbit IgG (BD Biosceinces San Jose, CA) at a dilution of 1/150 was used as a secondary antibody. Lightning-link conjugation kit (Innova Biosciences) was used to conjugate rabbit anti-human von-Willbrand factor antibody (Dako, Carpinteria, CA) to FITC and Anti-cardiac troponin T antibody (Abcam, Cambridge, MA) to PE. Cells were stained with lightning link-FITC conjugated vWF at a dilution of 1/10 and lightning link-PE conjugated Troponin T at a dilution 1/250. Isotype controls were used to define positive and negative populations. Labeled cells were analyzed using a MACSquant analyzer (Miltenyi Biotec, Auburn, CA). Dead cells and small particles were gated out using forward-scatter, side-scatter gating. FlowJo software (Ashland, OR) was used for fluorescence quantification.

### Cell Cycle

For cell cycle analysis, 100,000 cells were fixed with 70% ethanol, incubated for 60 minutes with RNAse A (Fisher Scientific Pittsburg, PA) and stained with propidium iodide, prior to running samples on a MACSquant analyzer (Miltenyi Biotec, Auburn, CA) and analyzing cell cycle progression using Flowjo software.

### RT-PCR

Cells were trypsinized and stored using Trizol reagent. Five hundred nanograms of RNA was isolated and made into cDNA using Superscript III protocol. RT-PCR was run using an IQ5 machine (Bio-rad, Hercules, CA). Beta actin was used as a housekeeping gene. Primer sequences are listed in S1 Table. The PCR conditions were: 94°C for 10 minutes, 94°C for 15 seconds, 52°C for 60 seconds, 72°C for 30 seconds for a total of 40 cycles. For microRNA experiments, RNA was converted to cDNA using the miScript II RT Kit (SABiosciences, Valencia CA), and then run on the human development and differentiation miScript plates (SABiosciences, Valencia CA). Individual primers for SNORD96a, SNORD72, hsa-miR-100-5p, and hsa-miR-99a-5p (SABiosciences, Valencia CA) were also used. The average expression of SNORD96a and SNORD72 was used as a housekeeping control. The PCR conditions for the microRNA arrays and individual microRNA assays were: 95°C for 15 minutes, 94°C for 15 seconds, 55°C for 60 seconds, 70°C for 30 seconds for a total of 40 cycles. MicroRNA expression data has been deposited in NCBI’s Gene Expression Omnibus [19] and is accessible through GEO series accession number GSE65795. MicroRNAs expressed at significantly different levels during simulated microgravity were analyzed using DIANA mirPATH software (Athens, Greece). DIANA mirpath software performs miRNA pathway analysis through hierarchical clustering of miRNAs and pathways based on their interaction levels comparing each set of microRNA targets to all known KEGG pathways [20]. Pathways significantly regulated by altered microRNAs were grouped according to KEGG pathway classifications.

### Tube Formation Assay

A 96-well plate was coated with basement membrane extract (50μL/well, Trevigen Gaithersburg, MD) 30 minutes prior to cells being plated on their surface at 20,000 cells per well. Cells were then incubated for 5 hours in EGM-2 media (Lonza Allendale, NJ) with 10% fetal bovine serum. Following incubation, cells were stained with Calcein AM (Fisher Scientific, Pittsburg, PA) and their ability to form capillary-like networks was measured. An EVOS microscope and Image PRO software was used to quantify tube formation.

### Statistics

For cell cycle, migration, flow cytometry, and microRNA RT-PCR profiling, a paired student’s t test was used. For RT-PCR and endothelial tube formation assay a student’s t test was used. Significance was p<0.05. Data is represented as the mean +/- standard error.

## Results

### Simulated microgravity increases growth factor expression without influencing cell migration

**Fig 1:**
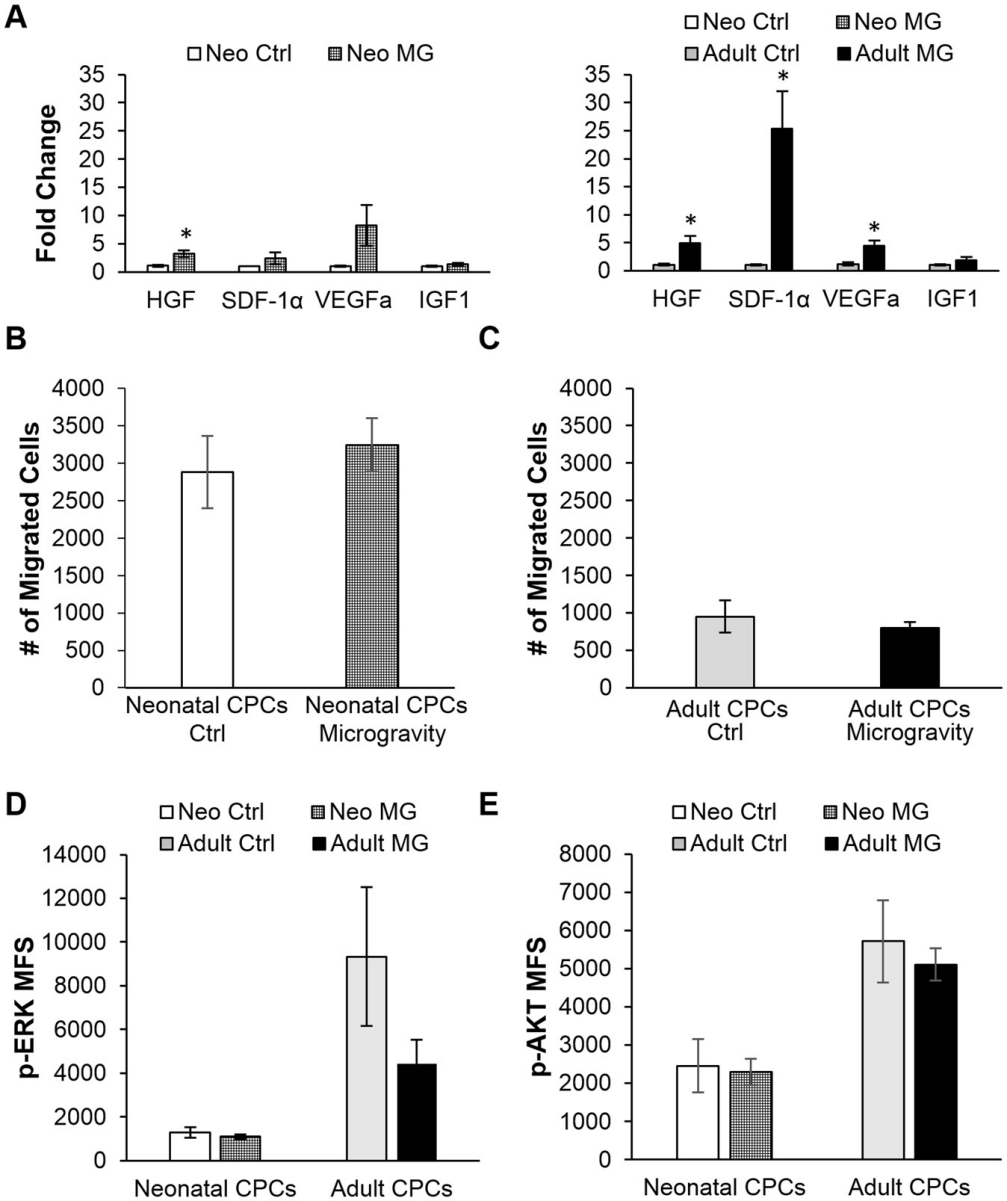
Cell Function after exposure to 6–7 days of simulated microgravity. A) Simulated microgravity significantly increased the expression of growth factors in both neonatal and adult CPCs by RT-PCR (n = 3, run in triplicate * = p<0.05). After simulated microgravity, neonatal and adult CPCs were plated in transwell plates to assess migration. Neither B) neonatal or C) adult CPCs demonstrated a change in the number of migrated cells after clinorotation. Phosphorylation of D) ERK and E) AKT was not significantly altered after simulated microgravity as shown by flow cytometry. Mean fluorescence shift (MFS) above secondary antibody alone is shown.

Isl-1+ cardiovascular progenitor cell (CPC) clones isolated from the heart of human neonates ≤1 month old and 57–75 year old adults [13] were exposed to simulated microgravity by culturing cells for 6–7 days in a 2D clinostat. The influence of simulated microgravity on the transcription of genes encoding factors with a paracrine effect, specifically the expression of growth factors including hepatocyte growth factor (HGF), serum-derived factor-1α (SDF-1α), vascular endothelial growth factor (VEGFa), and insulin-like growth factor-1 (IGF-1) was measured by RT-PCR. HGF, SDF-1α, VEGFa, and IGF-1 are growth factors that can be secreted by cardiac progenitors and can help enhance cardiac repair [21–25]. Both neonatal and adult cardiovascular progenitors demonstrated elevated expression of transcripts for HGF after simulated microgravity (Fig 1A). Interestingly, SDF-1α and VEGFa were more significantly elevated in adult CPCs (p<0.05).

Increased expression of paracrine factors could influence the ability of CPCs to migrate in response to growth factor stimulation. Therefore, cell migration was assessed using a transwell invasion assay. Neonatal and adult CPCs were cultured in simulated microgravity, then plated in the top transwell chamber. Normal growth medium supplemented with 100ng/ml SDF-1α was used as a chemoattractant. The number of migrated cells although higher at baseline in neonatal CPCs, was not significantly altered after exposure to simulated microgravity in either neonatal or adult CPCs (Fig 1B and 1C).

### ERK and AKT phosphorylation in CPCs is unchanged by exposure to simulated microgravity

Increased expression of growth factors could be a result of altered signaling in key pathways such as the MAPK signaling pathway. MAPK signaling regulates various cell functions such as proliferation, migration, and differentiation. Changes in MAPK signaling after simulated microgravity was assessed by comparing the level of extracellular signal-regulated kinase (ERK) phosphorylation by flow cytometry. Activation of the PI3k-AKT signaling pathway through phosphorylation of protein Kinase B (AKT), was also measured by flow cytometry. PI3k-AKT signaling is associated with increased proliferation and cell survival. After 6–7 days of simulated microgravity, the phosphorylation of ERK and AKT was not significantly altered in neonatal and adult CPCs (Fig 1D and 1E).

### Simulated microgravity increases cell number and transcripts for DNA repair genes in neonatal CPCs

**Fig 2:**
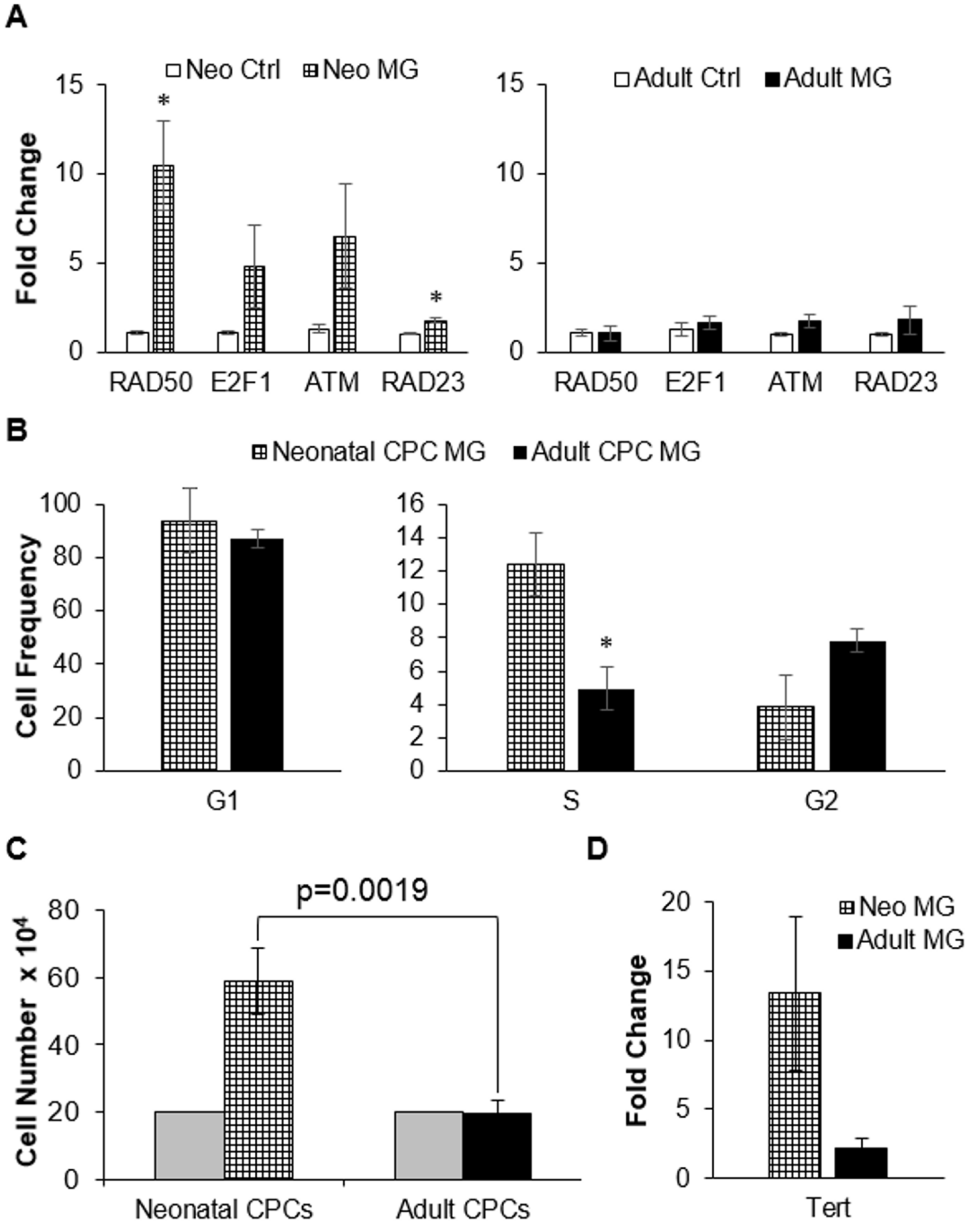
DNA repair and cell number after 6–7 days of simulated microgravity. A) DNA repair transcripts including RAD50, E2F1, ATM, RAD23 were elevated after simulated microgravity in neonatal (n = 4, run in triplicate, * = p<0.05) but not adult CPCs (n = 3, run in triplicate). B) A significantly higher frequency of neonatal CPCs entered the S phase of the cell cycle when compared with adult CPCs in simulated microgravity (12.4% vs 4.9%, p = 0.03). G1 phase and G2 phase did not differ significantly between neonatal and adult CPCs (G1 phase—93.3% vs 87.1%, p = 0.62 and G2 phase—3.9% vs 7.8%, p = 0.12 respectively). C) The average number of cells after 6–7 days of clinorotation was significantly higher in neonatal CPCs (5.89 x 10^4^, n = 12) when compared with adult CPCs (1.95 x 10^4^, n = 11). Number of cells seeded is shown in grey. D) The expression level of human telomerase reverse transcriptase was significantly elevated in neonatal (n = 4, run in triplicate) but not adult CPCs after simulated microgravity. Fold change above matched control is shown.

A unique feature of early stem cells is the high expression level of proteins that protect their DNA from damage. This allows the cells to divide and maintain genomic integrity. As stem cells age or differentiate, they lose this ability to effectively protect their DNA which can, over time, lead to cell senescence or death. After exposure to simulated microgravity, elevated expression of DNA repair transcripts including RAD50 (10.4 fold) and RAD23 (1.8 fold) was noted in neonatal CPCs (Fig 2A, n = 4, p<0.05). E2F1 and ATM were also elevated in neonatal CPCs, but not significantly (4.8 fold, p = 0.12 and 6.5 fold, p = 0.085 respectively). These changes in gene transcription were not present in adult CPCs (Fig 2A). Elevated transcription of DNA repair genes in neonatal CPCs after simulated microgravity was associated with an increased percentage of cells within the S phase of the cell cycle (12.4% vs 4.9%, p = 0.03, Fig 2B) and an increase in cell proliferation when compared with adult CPCs exposed to simulated microgravity. After 6–7 days in simulated microgravity, neonatal CPCs approximately tripled the number of cells seeded (58.9 x 10^4^ vs 20 x 10^4^ cells seeded) whereas adult CPCs did not increase in cell number (19.5 x 10^4^ vs 20 x 10^4^ cells seeded, Fig 2C). Some of the DNA repair proteins, for example RAD50, play a critical role in telomere maintenance [26, 27]. Therefore, we measured the expression level of telomerase reverse transcriptase (Tert), which is a protein responsible for maintaining telomere ends. After simulated microgravity, Tert was elevated 13.3 fold in neonatal CPCs (p = 0.034) whereas adult CPCs were only elevated 2.2 fold (p = 0.17, Fig 2D).

### Elevated expression of stemness-associated genes in neonatal CPCs after simulated microgravity

**Fig 3:**
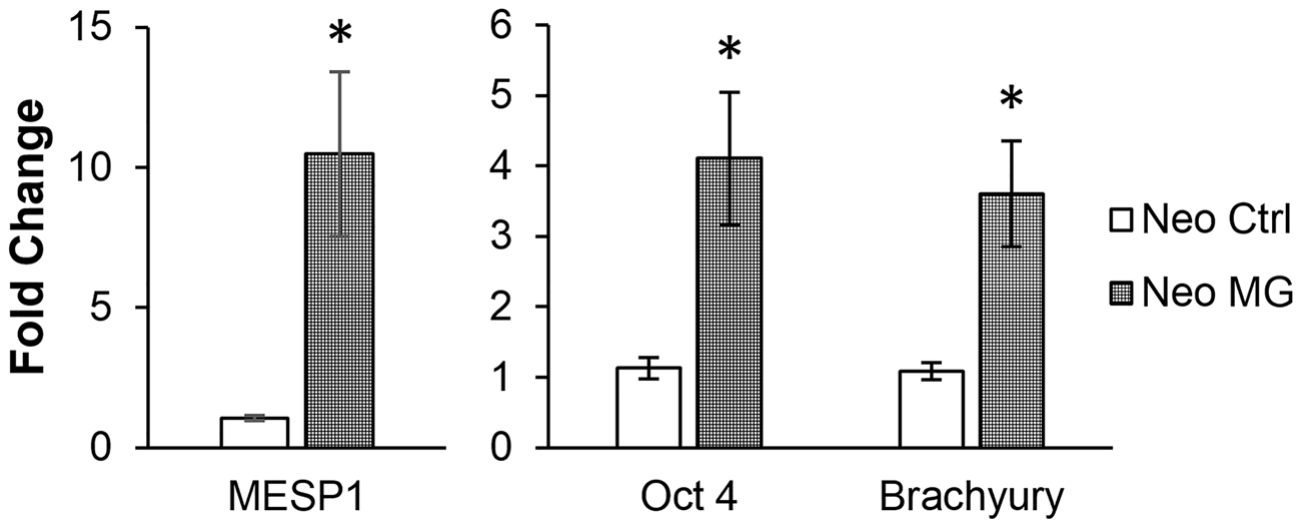
Transcripts associated with stemness are elevated in neonatal CPCs after 6–7 days of simulated microgravity. The expression of genes associated with early progenitor populations including MESP1, Oct-4, and Brachyury were significantly elevated in neonatal CPCs after simulated microgravity (n = 4, run in triplicate, p<0.05).

The increased expression of genes involved with DNA integrity after exposure to simulated microgravity may be the result of a shift in neonatal CPCs to a more undifferentiated state. This possibility was explored by assessing the level of expression of stemness-associated genes (Fig 3). After exposure to simulated microgravity, neonatal CPCs showed a significant increase in transcripts for early stem cell genes, including Mesoderm Posterior Protein 1 (MESP1, 10.48 Fold, p = 0.004). MESP1 is one of the earliest markers of cardiac stem cells [28]. Other stemness-associated genes were also elevated in expression, including Octamer-Binding Protein 4 (Oct-4, 5.5 Fold, p = 0.0016) and Brachyury (3.6 fold, p = 0.0032). Both Brachyury and Oct-4 are known to play a role in the induction of MESP1, early in the process of stem cell commitment to cardiac lineage [29, 30].

### MicroRNA profiling of neonatal CPCs after simulated microgravity

**Fig 4:**
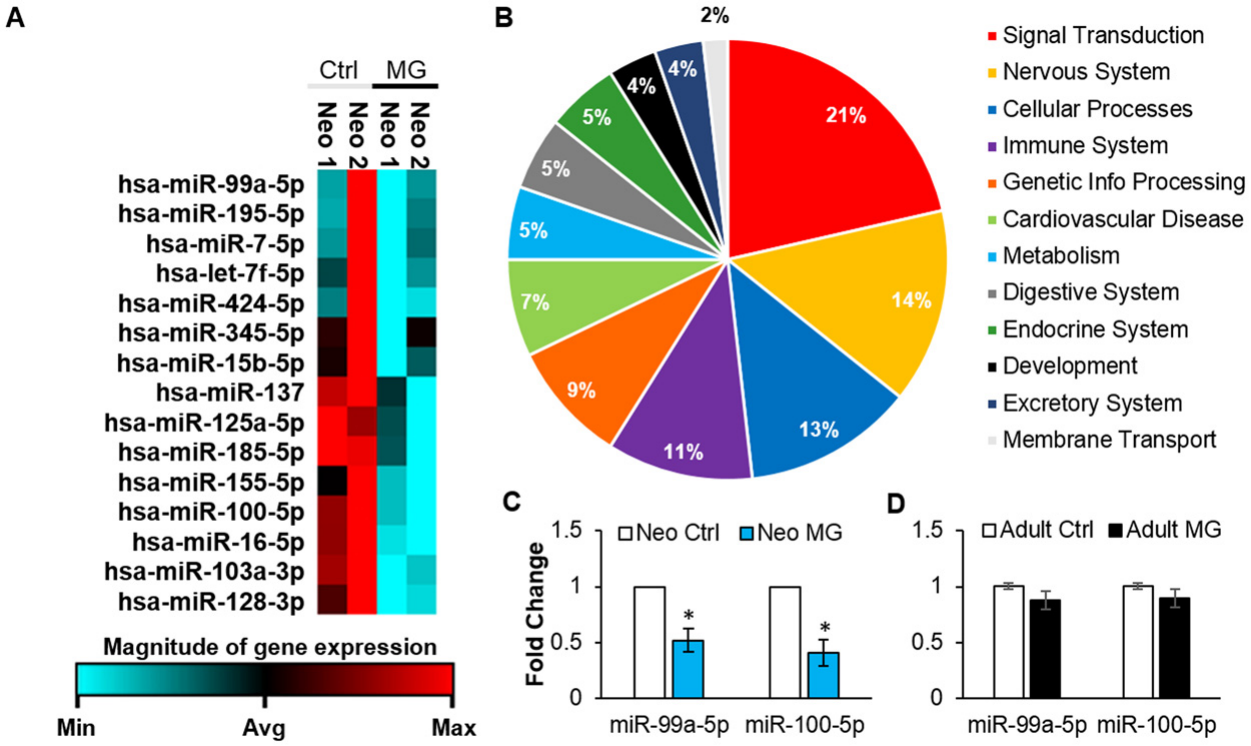
MicroRNA expression changes in neonatal CPCs after 6–7 days of simulated microgravity. A) Heat map of 15 microRNAs that were significantly altered in neonatal CPCs after simulated microgravity. Two representative neonatal CPC clones are shown above. Red color identifies maximum expression, black color represents average expression, blue color identifies microRNAs with minimum expression. Sets of co-regulated microRNAs were grouped together by RT^2^ Profiler PCR Array Data Analysis software (SABiosciences). B) MicroRNAs expressed at significantly different levels during simulated microgravity were analyzed using DIANA mirPATH software. Pathways significantly regulated by altered microRNAs were grouped according to KEGG pathway classifications, and the percentage of pathways in each category is displayed above. C) MicroRNA-100-5p and miR-99a-5p whose expression levels negatively correlate with stemness, were significantly downregulated after 6–7 days of simulated microgravity in neonatal CPCs (n = 3, p<0.05). D) Expression of microRNA-99a-5p and miR-100-5p were unchanged in adult CPCs after simulated microgravity (n = 3, run in triplicate).

MicroRNAs regulate gene expression through translational inhibition or degradation of mRNA. In stem cells, microRNAs can play a large role in stemness; the addition of a specific exogenous microRNA can change cell identity [31]. MicroRNA profiling was used for further assessment of cell stemness in neonatal CPCs after 6–7 days of simulated microgravity. RNA was isolated and microRNAs associated with cell development and differentiation were profiled by RT-PCR. In response to simulated microgravity the expression of 15 microRNAs were significantly altered (Fig 4A).

To better understand the impact of microRNA expression differences on neonatal CPCs, the 15 significantly altered microRNAs were analyzed using DIANA mirpath software v2.0. DIANA mirpath software performs miRNA pathway analysis through hierarchical clustering of miRNAs and pathways based on their interaction levels [20]. DIANA mirpath revealed 56 pathways (excluding pathways involved in other diseases) that were significantly regulated by altered microRNAs (S2 Table). Further classification of pathways was performed by using KEGG pathway database as a means of sorting pathways into broad categories. The percentage of pathways in each category is shown in Fig 4B. The largest percentage of pathways altered by simulated microgravity were involved in signal transduction (21%). Thirteen percent of pathways were involved in cellular processes (such as cell cycle and regulation of actin cytoskeleton) and nine percent were involved in genetic information processing.

Two of the 15 significantly altered microRNAs, microRNA-99a-5p and microRNA-100-5p which were significantly down-regulated in response to simulated microgravity (1.9 fold decrease, p = 0.04 and 2.4 fold decrease, p = 0.03 respectively, Fig 4C) play a critical role in dedifferentiation [32]. To confirm that this response is specific to neonatal CPCs, the expression level of microRNAs 100-5p and 99a-5p were measured in adult CPCs before and after simulated microgravity. Adult CPCs did not demonstrate a significant difference in the expression of microRNA-99a-5p (1.1 fold decrease, p = 0.16) and microRNA-100-5p (1.1 fold decrease, p = 0.21) before and after exposure to simulated microgravity (Fig 4D).

### Differentiation of adult CPCs in response to simulated microgravity

**Fig 5:**
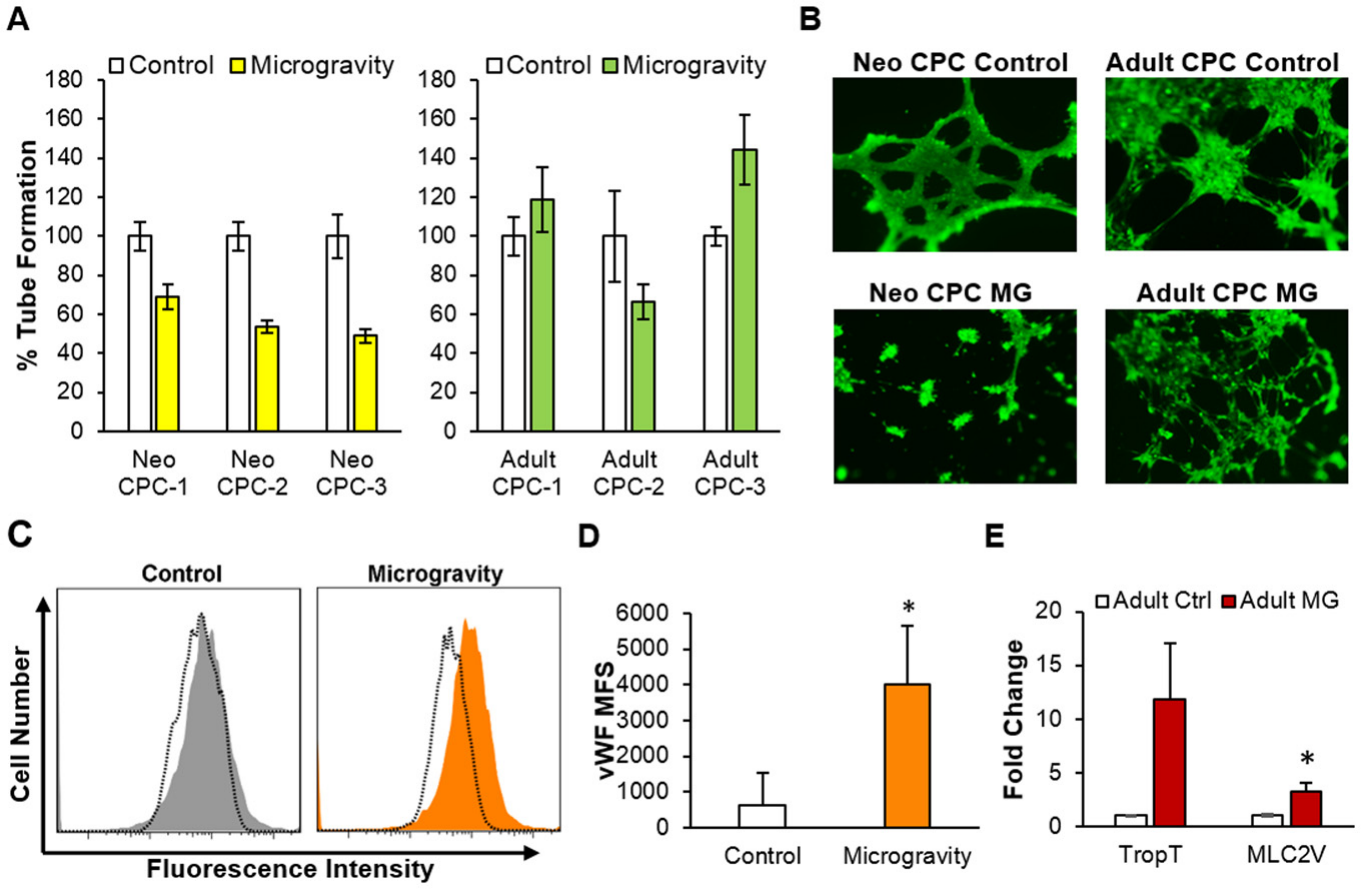
Cardiac progenitor cell differentiation after 6–7 days of simulated microgravity. A) Quantification of tube formation using Image pro plus software. Reduced tube formation was present in neonatal CPCs after simulated microgravity whereas 2 of 3 adult CPCs exhibited elevated tube formation after simulated microgravity. B) Representative images of tube formation in neonatal and adult CPC after 6–7 days of simulated microgravity. C) Representative flow cytometry histogram of increased von Willebrand Factor (vWF) expression in adult CPCs after simulated microgravity. Positive staining is represented by a colored histogram, secondary alone staining is represented as a dotted line. D) Quantification of von Willebrand factor protein expression in adult CPCs (n = 3, p = 0.047). Mean fluorescence shift above secondary antibody alone is shown above. E) Relative expression of genes associated with cardiac differentiation in adult CPCs after 6–7 days of simulated microgravity by RT-PCR. Myosin light chain 2 (MLC2V) an early indicator of cardiovascular differentiation was significantly elevated in adult CPCs (n = 3, run in triplicate P = 0.017).

CPC differentiation into both cardiovascular and endothelial cell lineages was measured after exposure to 6–7 days of simulated microgravity. A tube formation assay was used to assess endothelial differentiation. After simulated microgravity, neonatal CPCs had decreased tube formation (Fig 5A and 5B). Decreased tube formation in neonatal CPCs was complimented by a decreased expression level of von Willebrand Factor (vWF), an endothelial differentiation marker (1137 MFU decrease, n = 4, p = 0.17). In contrast, two of three adult CPCs showed an increase in tube formation after simulated microgravity (Fig 5A and 5B). This elevation in tube formation in adult CPCs after simulated microgravity was associated with a significant increase in the expression of vWF as determined by flow cytometry (3402 MFU increase, n = 3, p = 0.047, Fig 5C and 5D).

Cardiomyogenic differentiation was assessed by measuring the expression of mature cardiac-specific transcripts, troponin T (Trop T) and myosin light chain 2 (MLC2v) by RT-PCR. Gene expression levels for Trop T and MLC2V were elevated in adult CPCs after exposure to simulated microgravity (11.8 fold, p = 0.056 and 3.2 fold, p = 0.017 respectively, Fig 5E). Measurement of cardiac Troponin T protein expression by flow cytometry was used for further assessment of cardiomyogenic differentiation in adult CPCs. There was a small non-significant increase in the percentage of Troponin T positive cells (6.9%) in adult CPCs.

## Discussion

In this study, we assessed functional parameters, gene expression, and protein levels in stem cells that normally reside within heart after 6–7 days of 2D clinorotation. By using progenitors from both the neonatal and adult heart, we determined that age governs the functional outcome of cardiovascular stem cells exposed to simulated microgravity. Isl-1+ neonatal and adult cardiovascular progenitors exhibited similar responses to simulated microgravity in terms of cell migration and ERK/AKT activation, but opposing responses when comparing the effect of simulated microgravity on cell differentiation.

Studies performed in other laboratories using various cell types have reported that migration is one of the parameters that can be altered by exposure to 2D [33, 34] and 3D clinorotation [35, 36]. In our study, despite elevated levels of growth factor transcripts induced by simulated microgravity, cardiovascular progenitor cell migration was not altered. This is consistent with data reported by Versari et al (2007), who demonstrated that endothelial cell migration was not increased by exposure to simulated microgravity [37]. In our study, there was no evidence for activation of the ERK or AKT pathways in Isl-1+ cardiovascular progenitor cells, although mesenchymal stem cells have been reported to have reduced ERK [38] or AKT [4] phosphorylation levels as a consequence of simulated microgravity exposure. Simulated microgravity appears to have a cell-type dependent impact on activation and migration of stem cells.

Age-related differences significantly influenced differentiation under simulated microgravity in our study. Adult CPCs showed enhanced endothelial cell differentiation and elevated levels of von Willebrand factor after clinorotation. This is consistent with other studies in which mesenchymal stem cells demonstrated an increase in endothelial cell differentiation after simulated microgravity exposure [39]. Transcripts for MLC2v and Troponin T were elevated in adult Isl-1+ CPC, indicating that differentiation along the cardiomyocyte lineage and endothelial cell lineage is a functional outcome following culture of adult early cardiovascular progenitor cells under reduced gravity conditions.

Neonatal CPCs, on the other hand, did not form tubes effectively after clinorotation. This is consistent with other cell types, including osteoblasts and adipose-derived stem cells which show dedifferentiation and increased stemness with simulated microgravity exposure [9, 40]. In bone marrow stromal cells, dedifferentiation after simulated microgravity is a response that favors cell survival and improves regenerative capacity [41].

In an effort to gain further understanding regarding the mechanistic basis behind the differential impact of simulated microgravity on differentiation, we used microRNA profiling to study the epigenetic effects that simulated microgravity has on Isl-1+ neonatal cardiovascular progenitors. MicroRNAs are potent regulators of gene expression; one microRNA has the ability to target multiple genes in a given pathway. In other cell types, microRNA expression is significantly altered with exposure to simulated microgravity [42, 43] or real microgravity [44]. Girardi et al found that after 24 hours in simulated microgravity, the expression levels of 42 microRNAs were significantly changed in peripheral blood lymphocytes [45]. In our study, the expression of 15 microRNAs was significantly altered in neonatal CPCs after seven days of simulated microgravity. Interestingly, microRNAs-99a and miR-100 were among those which were significantly downregulated in neonatal, but not adult CPCs. MicroRNA-99a and miR-100 transcripts are undetectable in undifferentiated embryonic stem cells, but the level of expression increases with cell differentiation [46]. In animals with superior regenerative ability, such as zebrafish, microRNA-99a and miR-100 are strongly downregulated at the initiation of regeneration and result in dedifferentiation of existing cardiomyocytes [32].

Both of these microRNAs as well as microRNA-125a-5p (which was also significantly decreased with simulated microgravity) are downregulated during skin wound-healing [47]. MicroRNA-125a-5p represses the expression of pro-regenerative proteins such as Lin28[48]. Lin28 overexpression is associated with improved repair of tissues such as cartilage, bone, and mesenchyme [49]. Decreased expression of microRNA-125a-5p is associated with cell dedifferentiation [50] and in human embryonic stem cells antagonizing microRNA-125a-5p inhibits cell differentiation and upregulates pluripotency markers [51]. Several other microRNAs that were significantly downregulated in neonatal CPCs also positively correlate with cell differentiation including microRNA-424[52] and microRNA-137[46]. Furthermore, microRNAs that promote *in vitro* endothelial differentiation including microRNA-424[53], let-7f [54], and miR-155[55] were downregulated with simulated microgravity in neonatal CPCs.

The reduction in the expression of microRNAs that positively correlate with cell differentiation after simulated microgravity, although significant, are not of a magnitude to recapitulate embryonic stem cell phenotype. Many of these microRNAs are elevated anywhere from 40 to 1400 fold in neonatal CPCs when compared with human embryonic stem cells (S1 Fig) whereas, with simulated microgravity, microRNAs are decreased approximately 2 to 3 fold. Therefore, neonatal CPCs, when exposed to microgravity, have characteristics of dedifferentiated cells, but the microRNA expression profile is not equivalent to that of embryonic stem cells.

A unique characteristic of early stem cell populations is their high levels of DNA repair proteins and telomerase reverse transcriptase that play a role in maintaining DNA integrity over a large number of population doublings [56]. In our study, we found high levels of telomerase reverse transcriptase and DNA repair proteins in neonatal CPCs after simulated microgravity. In contrast to this, Kumari et al reported that, in human lymphocytes, exposure to clinorotation for 7 days reduced the expression of DNA repair proteins [57] and similarly, Sun et al reported reduced telomerase activity in MSCs cultured in a rotary cell culture system [58]. Our data demonstrates that elevated expression of DNA repair proteins in neonatal Isl-1+ CPCs with simulated microgravity is supported by microRNA expression differences. MicroRNA-195 which was significantly decreased with simulated microgravity (p = 0.0083) is predicted to inhibit RAD50 expression (using miRanda, miRDB, miRWalk, and Targetscan databases), and microRNA-185 which was also decreased with simulated microgravity (p = 0.028) is predicted to inhibit RAD23A expression (using miRanda, miRWalk, TargetScan databases).

Further evidence of neonatal CPC dedifferentiation, as a consequence of exposure to simulated microgravity, is the upregulation of stemness-associated genes MESP1, brachyury and Oct4. All three of these genes are characteristic of an early stem cell population and are expressed early in the process of cell commitment to cardiogenesis [28–30]. In our study there was a 4-fold increase in the expression of Oct-4. In neural stem cells, retroviral vector-induced expression of Oct-4 alone was enough to reprogram cells to a pluripotent state functionally similar to embryonic stem cells [59]. Oct4 is known to bind at the promoter of miR-137[60], a microRNA which inhibits cell stemness [61], this microRNA was also significantly decreased with simulated microgravity in neonatal CPCs.

When compared with the adult heart, the neonatal heart contains progenitors that have superior regenerative capacity [62]. During the neonatal window, the heart is a rich source of early cardiovascular progenitors and as an infant matures, the heart increases in size while the proportion of early stem cells decreases [63, 64]. In this study, we were able to manipulate the process whereby neonatal cardiovascular progenitor cells activate a conserved regenerative microRNA program. In other cell types, a simulated microgravity-induced shift towards an undifferentiated state enhanced the repair potential of cells [41]. Whether, in our study, this change in cell phenotype displayed by neonatal cardiovascular progenitors is advantageous, long-lasting, and provides a cell type with greater regenerative capacity has yet to be determined. Future studies planned on the International Space Station will determine whether a similar effect occurs in the space environment or if this phenomenon is induced by shear stress forces that may influence gene expression under simulated microgravity conditions.

## Supporting Information

S1 Fig Expression levels of microRNAs positively associated with cell differentiation. (TIF)

S1 Table Primer sequences used for RT-PCR. (PDF)

S2 Table Pathway analysis associated with microRNAs that were differentially regulated in neonatal CPCs after 7 days of microgravity exposure. (PDF)

## References

[1] White RJ, Averner M. Humans in space. Nature. 2001;409(6823):1115–8. 11234026 10.1038/35059243

[2] Bradamante S, Barenghi L, Maier JA. Stem Cells toward the Future: The Space Challenge. Life. 2014;4(2):267–80. 10.3390/life4020267 25370198 PMC4187162

[3] Huang Y, Dai ZQ, Ling SK, Zhang HY, Wan YM, Li YH. Gravity, a regulation factor in the differentiation of rat bone marrow mesenchymal stem cells. J Biomed Sci. 2009;16:87 10.1186/1423-0127-16-87 19772591 PMC2754420

[4] Dai Z-Q, Wang R, Ling S, Wan Y, Li Y. Simulated microgravity inhibits the proliferation and osteogenesis of rat bone marrow mesenchymal stem cells. Cell proliferation. 2007;40(5):671–84. 17877609 10.1111/j.1365-2184.2007.00461.x PMC6496371

[5] Zayzafoon M, Gathings WE, McDonald JM. Modeled microgravity inhibits osteogenic differentiation of human mesenchymal stem cells and increases adipogenesis. Endocrinology. 2004;145(5):2421–32. 14749352 10.1210/en.2003-1156

[6] Saxena R, Pan G, McDONALD J. Osteoblast and osteoclast differentiation in modeled microgravity. Annals of the New York Academy of Sciences. 2007;1116(1):494–8. 17656572 10.1196/annals.1402.033

[7] Hwang Y-S, Cho J, Tay F, Heng JY, Ho R, Kazarian SG, et al The use of murine embryonic stem cells, alginate encapsulation, and rotary microgravity bioreactor in bone tissue engineering. Biomaterials. 2009;30(4):499–507. 10.1016/j.biomaterials.2008.07.028 18977027

[8] McGuckin C, Forraz N, Baradez MO, Navran S, Zhao J, Urban R, et al Production of stem cells with embryonic characteristics from human umbilical cord blood. Cell proliferation. 2005;38(4):245–55. 16098183 10.1111/j.1365-2184.2005.00346.x PMC6496335

[9] Zhang S, Liu P, Chen L, Wang Y, Wang Z, Zhang B. The effects of spheroid formation of adipose-derived stem cells in a microgravity bioreactor on stemness properties and therapeutic potential. Biomaterials. 2015;41:15–25. 10.1016/j.biomaterials.2014.11.019 25522961

[10] Talbot NC, Caperna TJ, Blomberg L, Graninger PG, Stodieck LS. The effects of space flight and microgravity on the growth and differentiation of PICM-19 pig liver stem cells. In Vitro Cellular & Developmental Biology-Animal. 2010;46(6):502–15. 20333478 10.1007/s11626-010-9302-6

[11] Pisanu ME, Noto A, De Vitis C, Masiello MG, Coluccia P, Proietti S, et al Lung cancer stem cell lose their stemness default state after exposure to microgravity. BioMed research international. 2014;2014. 10.1155/2014/470253 PMC4170742 25276790

[12] Simpson DL, Mishra R, Sharma S, Goh SK, Deshmukh S, Kaushal S. A strong regenerative ability of cardiac stem cells derived from neonatal hearts. Circulation. 2012;126(11 Suppl 1):S46–53. Epub 2012/09/22. 10.1161/CIRCULATIONAHA.111.084699 . 22965993 PMC4801017

[13] Fuentes TI, Appleby N, Tsay E, Martinez JJ, Bailey L, Hasaniya N, et al Human Neonatal Cardiovascular Progenitors: Unlocking the Secret to Regenerative Ability. PLoS ONE. 2013;8(10):e77464 10.1371/journal.pone.0077464 24204836 PMC3810469

[14] Smits AM, van Vliet P, Metz CH, Korfage T, Sluijter JP, Doevendans PA, et al Human cardiomyocyte progenitor cells differentiate into functional mature cardiomyocytes: an in vitro model for studying human cardiac physiology and pathophysiology. Nat Protoc. 2009;4(2):232–43. Epub 2009/02/07. 10.1038/nprot.2008.229 . 19197267

[15] Krutzik PO, Nolan GP. Intracellular phospho-protein staining techniques for flow cytometry: Monitoring single cell signaling events. Cytometry Part A. 2003;55(2):61–70. 10.1002/cyto.a.10072 14505311

[16] Abrahamsen I, Lorens JB. Evaluating Extracellular Matrix influence on adherent cell signaling by Cold Trypsin Phosphorylation-specific Flow Cytometry. BMC cell biology. 2013;14(1):36. 23957395 10.1186/1471-2121-14-36 PMC3751818

[17] Jun HS, Lee YM, Song KD, Mansfield BC, Chou JY. G-CSF improves murine G6PC3-deficient neutrophil function by modulating apoptosis and energy homeostasis. Blood. 2011;117(14):3881–92. 10.1182/blood-2010-08-302059 21292774 PMC3083300

[18] Krutzik PO, Irish JM, Nolan GP, Perez OD. Analysis of protein phosphorylation and cellular signaling events by flow cytometry: techniques and clinical applications. Clinical Immunology. 2004;110(3):206–21. 15047199 10.1016/j.clim.2003.11.009

[19] Edgar R, Domrachev M, Lash AE. Gene Expression Omnibus: NCBI gene expression and hybridization array data repository. Nucleic acids research. 2002;30(1):207–10. 11752295 10.1093/nar/30.1.207 PMC99122

[20] Vlachos IS, Kostoulas N, Vergoulis T, Georgakilas G, Reczko M, Maragkakis M, et al DIANA miRPath v. 2.0: investigating the combinatorial effect of microRNAs in pathways. Nucleic acids research. 2012;40(W1):W498–W504. 22649059 10.1093/nar/gks494 PMC3394305

[21] Chimenti I, Smith RR, Li TS, Gerstenblith G, Messina E, Giacomello A, et al Relative roles of direct regeneration versus paracrine effects of human cardiosphere-derived cells transplanted into infarcted mice. Circ Res. 2010;106(5):971–80. 10.1161/CIRCRESAHA.109.210682 . 20110532 PMC4317351

[22] Hu X, Dai S, Wu WJ, Tan W, Zhu X, Mu J, et al Stromal cell derived factor-1 alpha confers protection against myocardial ischemia/reperfusion injury: role of the cardiac stromal cell derived factor-1 alpha CXCR4 axis. Circulation. 2007;116(6):654–63. 10.1161/CIRCULATIONAHA.106.672451 17646584 PMC3640445

[23] Cheng K, Malliaras K, Smith RR, Shen D, Sun B, Blusztajn A, et al Human cardiosphere-derived cells from advanced heart failure patients exhibit augmented functional potency in myocardial repair. JACC Heart failure. 2014;2(1):49–61. 10.1016/j.jchf.2013.08.008 24511463 PMC3914736

[24] Linke A, Muller P, Nurzynska D, Casarsa C, Torella D, Nascimbene A, et al Stem cells in the dog heart are self-renewing, clonogenic, and multipotent and regenerate infarcted myocardium, improving cardiac function. Proc Natl Acad Sci U S A. 2005;102(25):8966–71. 10.1073/pnas.0502678102 15951423 PMC1157041

[25] Tang J, Wang J, Kong X, Yang J, Guo L, Zheng F, et al Vascular endothelial growth factor promotes cardiac stem cell migration via the PI3K/Akt pathway. Exp Cell Res. 2009;315(20):3521–31. 10.1016/j.yexcr.2009.09.026 . 19800880

[26] Vannier JB, Depeiges A, White C, Gallego ME. Two roles for Rad50 in telomere maintenance. The EMBO journal. 2006;25(19):4577–85. 16990794 10.1038/sj.emboj.7601345 PMC1589983

[27] Gallego ME, White CI. RAD50 function is essential for telomere maintenance in Arabidopsis. Proceedings of the National Academy of Sciences. 2001;98(4):1711–6. 10.1073/pnas.98.4.1711 PMC29322 11172016

[28] Bondue A, Tännler S, Chiapparo G, Chabab S, Ramialison M, Paulissen C, et al Defining the earliest step of cardiovascular progenitor specification during embryonic stem cell differentiation. The Journal of cell biology. 2011;192(5):751–65. 10.1083/jcb.201007063 21383076 PMC3051813

[29] David R, Jarsch VB, Schwarz F, Nathan P, Gegg M, Lickert H, et al Induction of MesP1 by Brachyury (T) generates the common multipotent cardiovascular stem cell. Cardiovascular research. 2011;92(1):115–22. 10.1093/cvr/cvr158 21632880

[30] Li Y, Yu W, Cooney AJ, Schwartz RJ, Liu Y. Brief Report: Oct4 and Canonical Wnt Signaling Regulate the Cardiac Lineage Factor Mesp1 Through a Tcf/Lef-Oct4 Composite Element. Stem Cells. 2013;31(6):1213–7. 10.1002/stem.1362 23417899

[31] Lin S-L, Ying S-Y. Mechanism and method for generating tumor-free iPS cells using intronic microRNA miR-302 induction MicroRNA Protocols: Springer; 2013 p. 295–312. 10.1007/978-1-62703-083-0_23 23007517

[32] Aguirre A, Montserrat N, Zachiggna S, Nivet E, Hishida T, Krause MN, et al In Vivo Activation of a Conserved MicroRNA Program Induces Mammalian Heart Regeneration. Cell Stem Cell. 2014;15(5):589–604. 10.1016/j.stem.2014.10.003 25517466 PMC4270016

[33] Gershovich J, Buravkova L. Morphofunctional status and osteogenic differentiation potential of human mesenchymal stromal precursor cells during in vitro modeling of microgravity effects. Bulletin of experimental biology and medicine. 2007;144(4):608–13. 18642723 10.1007/s10517-007-0387-1

[34] Plett PA, Abonour R, Frankovitz SM, Orschell CM. Impact of modeled microgravity on migration, differentiation, and cell cycle control of primitive human hematopoietic progenitor cells. Experimental hematology. 2004;32(8):773–81. 15308329 10.1016/j.exphem.2004.03.014

[35] Mitsuhara T, Takeda M, Yamaguchi S, Manabe T, Matsumoto M, Kawahara Y, et al Simulated microgravity facilitates cell migration and neuroprotection after bone marrow stromal cell transplantation in spinal cord injury. Stem Cell Res Ther. 2013;4(2):35 10.1186/scrt184 23548163 PMC3706926

[36] Espinosa-Jeffrey A, Paez PM, Cheli VT, Spreuer V, Wanner I, de Vellis J. Impact of Simulated Microgravity on Oligodendrocyte Development: Implications for Central Nervous System Repair. PloS one. 2013;8(12):e76963 10.1371/journal.pone.0076963 24324574 PMC3850904

[37] Versari S, Villa A, Bradamante S, Maier JA. Alterations of the actin cytoskeleton and increased nitric oxide synthesis are common features in human primary endothelial cell response to changes in gravity. Biochimica et Biophysica Acta (BBA)-Molecular Cell Research. 2007;1773(11):1645–52. 17609119 10.1016/j.bbamcr.2007.05.014

[38] Meyers VE, Zayzafoon M, Gonda SR, Gathings WE, McDonald JM. Modeled microgravity disrupts collagen I/integrin signaling during osteoblastic differentiation of human mesenchymal stem cells. Journal of cellular biochemistry. 2004;93(4):697–707. 15660414 10.1002/jcb.20229

[39] Zhang X, Nan Y, Wang H, Chen J, Wang N, Xie J, et al Model microgravity enhances endothelium differentiation of mesenchymal stem cells. Naturwissenschaften. 2013;100(2):125–33. 10.1007/s00114-012-1002-5 23229853

[40] Ontiveros C, McCabe LR. Simulated microgravity suppresses osteoblast phenotype, Runx2 levels and AP-1 transactivation. Journal of cellular biochemistry. 2003;88(3):427–37. 12532319 10.1002/jcb.10410

[41] Yuge L, Sasaki A, Kawahara Y, Wu S-l, Matsumoto M, Manabe T, et al Simulated microgravity maintains the undifferentiated state and enhances the neural repair potential of bone marrow stromal cells. Stem cells and development. 2010;20(5):893–900. 10.1089/scd.2010.0294 20828292

[42] Allen DL, Bandstra ER, Harrison BC, Thorng S, Stodieck LS, Kostenuik PJ, et al Effects of spaceflight on murine skeletal muscle gene expression. Journal of Applied Physiology. 2009;106(2):582–95. 10.1152/japplphysiol.90780.2008 19074574 PMC2644242

[43] Mangala LS, Zhang Y, He Z, Emami K, Ramesh GT, Story M, et al Effects of simulated microgravity on expression profile of microRNA in human lymphoblastoid cells. Journal of Biological Chemistry. 2011;286(37):32483–90. 10.1074/jbc.M111.267765 21775437 PMC3173213

[44] Versari S, Longinotti G, Barenghi L, Maier JAM, Bradamante S. The challenging environment on board the International Space Station affects endothelial cell function by triggering oxidative stress through thioredoxin interacting protein overexpression: the ESA-SPHINX experiment. The FASEB Journal. 2013;27(11):4466–75. 23913861 10.1096/fj.13-229195

[45] Girardi C, De Pittà C, Casara S, Calura E, Romualdi C, Celotti L, et al Integration analysis of microRNA and mRNA expression profiles in human peripheral blood lymphocytes cultured in modeled microgravity. BioMed Research International. 2014;2014:16 10.1155/2014/296747 PMC4090438 25045661

[46] Tarantino C, Paolella G, Cozzuto L, Minopoli G, Pastore L, Parisi S, et al miRNA 34a, 100, and 137 modulate differentiation of mouse embryonic stem cells. The FASEB Journal. 2010;24(9):3255–63. 20439489 10.1096/fj.09-152207

[47] Jin Y, Tymen SD, Chen D, Fang ZJ, Zhao Y, Dragas D, et al MicroRNA-99 family targets AKT/mTOR signaling pathway in dermal wound healing. PloS one. 2013;8(5):e64434 10.1371/journal.pone.0064434 23724047 PMC3665798

[48] Zhong X, Li N, Liang S, Huang Q, Coukos G, Zhang L. Identification of microRNAs regulating reprogramming factor LIN28 in embryonic stem cells and cancer cells. Journal of Biological Chemistry. 2010;285(53):41961–71. 10.1074/jbc.M110.169607 20947512 PMC3009922

[49] Shyh-Chang N, Zhu H, De Soysa TY, Shinoda G, Seligson MT, Tsanov KM, et al Lin28 enhances tissue repair by reprogramming cellular metabolism. Cell. 2013;155(4):778–92. 10.1016/j.cell.2013.09.059 24209617 PMC3917449

[50] Zhu W-Y, Luo B, An J-y, He J-y, Chen D-d, Xu L-Y, et al Differential Expression of miR-125a-5p and let-7e Predicts the Progression and Prognosis of Non-Small Cell Lung Cancer. Cancer investigation. 2014;32(8):394–401. 10.3109/07357907.2014.922569 24945821

[51] Boissart C, Nissan X, Giraud-Triboult K, Peschanski M, Benchoua A. miR-125 potentiates early neural specification of human embryonic stem cells. Development. 2012;139(7):1247–57. 10.1242/dev.073627 22357933

[52] Rosa A, Ballarino M, Sorrentino A, Sthandier O, De Angelis F, Marchioni M, et al The interplay between the master transcription factor PU. 1 and miR-424 regulates human monocyte/macrophage differentiation. Proceedings of the National Academy of Sciences. 2007;104(50):19849–54. 10.1073/pnas.0706963104 PMC2148386 18056638

[53] Ghosh G, Subramanian IV, Adhikari N, Zhang X, Joshi HP, Basi D, et al Hypoxia-induced microRNA-424 expression in human endothelial cells regulates HIF-α isoforms and promotes angiogenesis. The Journal of clinical investigation. 2010;120(11):4141–54. 10.1172/JCI42980 20972335 PMC2964978

[54] Kuehbacher A, Urbich C, Zeiher AM, Dimmeler S. Role of Dicer and Drosha for endothelial microRNA expression and angiogenesis. Circulation research. 2007;101(1):59–68. 17540974 10.1161/CIRCRESAHA.107.153916

[55] Kong W, He L, Richards E, Challa S, Xu C, Permuth-Wey J, et al Upregulation of miRNA-155 promotes tumour angiogenesis by targeting VHL and is associated with poor prognosis and triple-negative breast cancer. Oncogene. 2013;33(6):679–89. 10.1038/onc.2012.636 23353819 PMC3925335

[56] Zeng X. Human embryonic stem cells: mechanisms to escape replicative senescence? Stem cell reviews. 2007;3(4):270–9. 18026912 10.1007/s12015-007-9005-x

[57] Kumari R, Singh KP, DuMond JW. Simulated microgravity decreases DNA repair capacity and induces DNA damage in human lymphocytes. Journal of cellular biochemistry. 2009;107(4):723–31. 10.1002/jcb.22171 19415677

[58] Sun L, Gan B, Fan Y, Xie T, Hu Q, Zhuang F. Simulated microgravity alters multipotential differentiation of rat mesenchymal stem cells in association with reduced telomerase activity. Acta Astronautica. 2008;63(7):968–73.

[59] Kim JB, Sebastiano V, Wu G, Araúzo-Bravo MJ, Sasse P, Gentile L, et al Oct4-induced pluripotency in adult neural stem cells. cell. 2009;136(3):411–9. 10.1016/j.cell.2009.01.023 19203577

[60] Boyer LA, Lee TI, Cole MF, Johnstone SE, Levine SS, Zucker JP, et al Core transcriptional regulatory circuitry in human embryonic stem cells. Cell. 2005;122(6):947–56. 16153702 10.1016/j.cell.2005.08.020 PMC3006442

[61] Bier A, Giladi N, Kronfeld N, Lee HK, Cazacu S, Finniss S, et al MicroRNA-137 is downregulated in glioblastoma and inhibits the stemness of glioma stem cells by targeting RTVP-1. Oncotarget. 2013;4(5):665–76. 23714687 10.18632/oncotarget.928 PMC3742828

[62] Jesty SA, Steffey MA, Lee FK, Breitbach M, Hesse M, Reining S, et al c-kit+ precursors support postinfarction myogenesis in the neonatal, but not adult, heart. Proc Natl Acad Sci U S A. 2012;109(33):13380–5. Epub 2012/08/01. 10.1073/pnas.1208114109 22847442 PMC3421216

[63] Amir G, Ma X, Reddy VM, Hanley FL, Reinhartz O, Ramamoorthy C, et al Dynamics of human myocardial progenitor cell populations in the neonatal period. Ann Thorac Surg. 2008;86(4):1311–9. Epub 2008/09/23. 10.1016/j.athoracsur.2008.06.058 . 18805183

[64] Mishra R, Vijayan K, Colletti EJ, Harrington DA, Matthiesen TS, Simpson D, et al Characterization and functionality of cardiac progenitor cells in congenital heart patients. Circulation. 2011;123(4):364–73. Epub 2011/01/19. 10.1161/CIRCULATIONAHA.110.971622 21242485 PMC3320857

