# Supplementary figures and images for: Simulated Microgravity Exerts an Age-Dependent Effect on the Differentiation of Cardiovascular Progenitors Isolated from the Human Heart
Source: PLoS One. 2015 Jul 10;10(7):e0132378. doi: 10.1371/journal.pone.0132378 (PMC4498633; doi:10.1371/journal.pone.0132378)

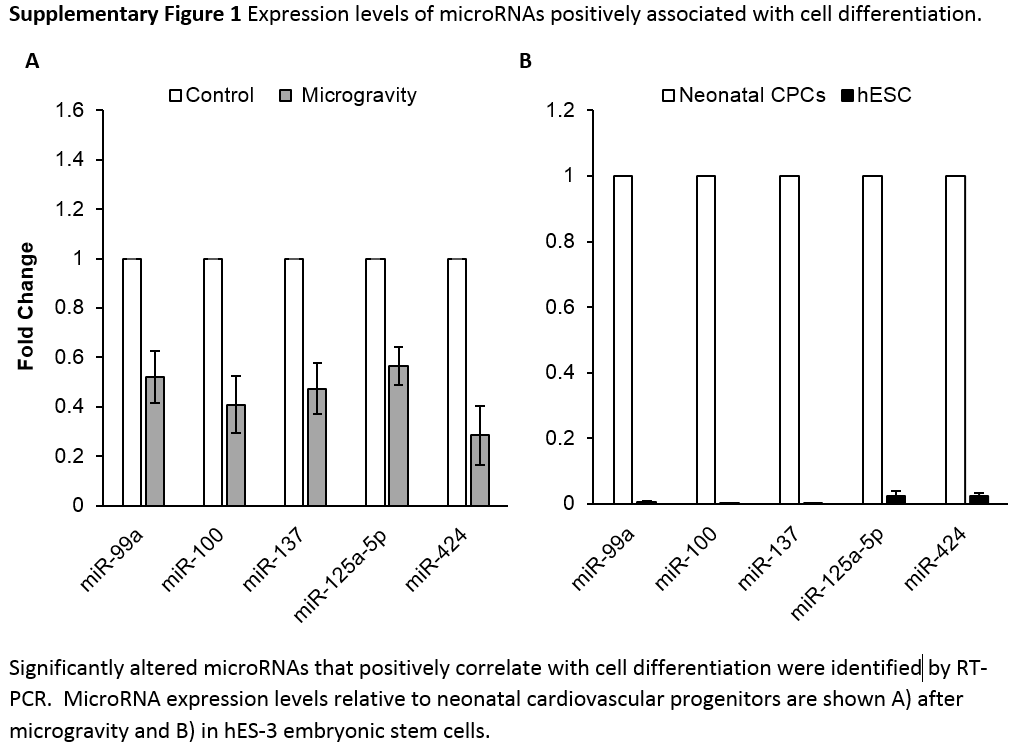

Supplement: S1 Fig — (TIF) [file pone.0132378.s001.tif]
