# Supplementary material for: Simulated Microgravity Exerts an Age-Dependent Effect on the Differentiation of Cardiovascular Progenitors Isolated from the Human Heart
Source: PLoS One. 2015 Jul 10;10(7):e0132378. doi: 10.1371/journal.pone.0132378 (PMC4498633; doi:10.1371/journal.pone.0132378)
Supplement: S1 Table — (PDF) [file pone.0132378.s002.pdf]

Table S1 – Primer Sequences used for RT-PCR

**Gene**	**Forward Primer**	**Reverse Primer**
ATM	GGGCGAGCCGCAAACGCTAA	TTCGGCCCGTCGGAGCAAAC
β-Actin	TTTGAATGATGAGCCTTCGTCCCC	GGTCTCAAGTCAGTGTACAGGTAAGC
Brachyury	ACTGGATGAAGGCTCCCGTCTCCTT	CCAAGGCTGGACCAATTGTCATGGG
CD31	TGTTGACATGAAGAGCCTGC	ACAGTTGACCCTCACGATCC
E2F1	GACCATCAGTACCTGGCCGAGAG	GACGACACCGTCAGCCGAGTG
HGF	CACGAACACAGCTTTTTGCC	TGATCCCAGCGCTGACAAAT
hTERT	AGAGTGTCTGGAGCAAGTTGC	CGTAGTCCATGTTCACAATCG
IGF1	CAGAGCAGATAGAGCCTGCG	CAGGTAACTCGTGCAGAGCA
MESP1	CGCTATATCGGCCACCTGTC	GGCATCCAGGTCTCCAACAG
MLC-2v	TATTGGAACATGGCCTCTGGAT	GGTGCTGAAGGCTGATTACGTT
MYC	AAGACAGCGGCAGCCCGAAC	TGGGCGAGCTGCTGTCGTTG
Oct 4	AACCTGGAGTTTGTGCCAGGGTTT	TGAACTTCACCTTCCCTCCAACCA
RAD23	GTATCGGAGCAGCCGGCCAC	TCCCCAGGGGGCTCGTTCAG
RAD50	CTACGGCTTTGCGTCCCCGG	ACACCAGCTGCTTTCCCCGC
SDF	CTACAGATGCCCATGCCGAT	GTGGGTCTAGCGGAAAGTCC
TROP T	GTGGGAAGAGGCAGACTGAG	ATAGATGCTCTGCCACAGC
VEGFa	CAGCGAAAGCGACAGGGGCA	GCTGGAGCACTGTCTGCGCA
