# Supplementary material for: Simulated Microgravity Exerts an Age-Dependent Effect on the Differentiation of Cardiovascular Progenitors Isolated from the Human Heart
Source: PLoS One. 2015 Jul 10;10(7):e0132378. doi: 10.1371/journal.pone.0132378 (PMC4498633; doi:10.1371/journal.pone.0132378)
Supplement: S2 Table — (PDF) [file pone.0132378.s003.pdf]

Table S2 - Pathway analysis associated with microRNAs that were differentially regulated in neonatal CPCs after 7 days of microgravity exposure.

KEGG pathway	p-value	# of miRNAs in Pathway	# of Genes Regulated by miRNAs
MAPK signaling pathway	2.6E-30	15	109
Insulin signaling pathway	1.6E-27	15	64
Neurotrophin signaling pathway	8.6E-24	15	57
mTOR signaling pathway	2.8E-22	15	36
ErbB signaling pathway	7.2E-19	15	44
PI3K-Akt signaling pathway	1.3E-16	15	121
Focal adhesion	2.9E-16	15	79
GnRH signaling pathway	7.0E-14	13	40
Ubiquitin mediated proteolysis	6.8E-13	12	56
Wnt signaling pathway	2.5E-12	15	60
T cell receptor signaling pathway	4.0E-11	13	44
Dorso-ventral axis formation	1.6E-10	13	17
Gap junction	1.8E-10	13	37
Hypertrophic cardiomyopathy (HCM)	2.0E-10	12	35
Dilated cardiomyopathy	4.9E-10	12	37
Endocytosis	8.3E-10	14	73
B cell receptor signaling pathway	2.5E-09	13	32
Fatty acid biosynthesis	3.1E-09	7	3
p53 signaling pathway	6.9E-09	12	30
VEGF signaling pathway	1.2E-08	13	28
Progesterone-mediated oocyte maturation	5.4E-08	13	34
Fc epsilon RI signaling pathway	2.1E-07	13	29
Long-term potentiation	2.2E-07	15	28
HIF-1 signaling pathway	2.5E-07	15	42
Lysine degradation	2.6E-07	13	21
Toll-like receptor signaling pathway	5.8E-07	13	41
ABC transporters	6.6E-07	12	19
Notch signaling pathway	7.0E-07	13	20
Fc gamma R-mediated phagocytosis	3.2E-06	13	35
Adipocytokine signaling pathway	7.2E-06	14	26
Hedgehog signaling pathway	2.4E-05	11	20
Long-term depression	6.5E-05	13	28
Regulation of actin cytoskeleton	8.2E-05	13	70
Axon guidance	0.0001	13	48
Viral myocarditis	0.0002	12	25
Chemokine signaling pathway	0.0002	15	59
Gastric acid secretion	0.0002	12	27
Calcium signaling pathway	0.0002	12	57
Arrhythmogenic right ventricular cardiomyopathy (ARVC)	0.0003	12	29
Glycosaminoglycan biosynthesis - heparan sulfate / heparin	0.0012	11	11
Osteoclast differentiation	0.0014	13	42
Dopaminergic synapse	0.0027	13	43

Retrograde endocannabinoid signaling	0.0027	13	38
TGF-beta signaling pathway	0.0049	14	27
Aldosterone-regulated sodium reabsorption	0.0072	13	14
Vasopressin-regulated water reabsorption	0.0074	10	15
Glutamatergic synapse	0.0079	13	38
RNA degradation	0.0167	13	23
Apoptosis	0.0172	12	30
Protein processing in endoplasmic reticulum	0.0206	12	53
Basal transcription factors	0.0258	12	15
Protein digestion and absorption	0.0317	12	27
Salivary secretion	0.0422	11	27
Cholinergic synapse	0.0479	13	38
mRNA surveillance pathway	0.0491	12	28
Adherens junction	0.0491	13	26
